# Quercetin Completely Ameliorates Hypoxia–Reoxygenation-Induced Pathophysiology Severity in NY1DD Transgenic Sickle Mice: Intrinsic Mild Steady State Pathophysiology of the Disease in NY1DD Is Also Reversed

**DOI:** 10.3390/biom11101473

**Published:** 2021-10-06

**Authors:** Sangeetha Thangaswamy, Craig A. Branch, Kamalakar Ambadipudi, Seetharama A. Acharya

**Affiliations:** 1 Division of Hematology, Department of Medicine, Montefiore Medical Center, Albert Einstein College of Medicine, 1300 Morris Park Avenue, Bronx, NY 10461, USA; sangeethaphd@gmail.com; 2 Magnetic Resonance Research Center, Department of Radiology, Albert Einstein College of Medicine, 1300 Morris Park Avenue, Bronx, NY 10461, USA; craig.branch@einsteinmed.org (C.A.B.); Kamal.ambad@einsteinmed.org (K.A.)

**Keywords:** hypoxia reoxygenation, blood flow, inflammation, leukocyte, quercetin, high oxygen affinity RBC, transgenic sickle mice, cremaster postcapillary venules

## Abstract

The vaso-occlusive crisis (VOC) is a major complication of sickle cell disease (SCD); thus, strategies to ameliorate vaso-occlusive episodes are greatly needed. We evaluated the therapeutic benefits of quercetin in a SCD transgenic sickle mouse model. This disease model exhibited very mild disease pathophysiology in the steady state. The severity of the disease in the NY1DD mouse was amplified by subjecting mice to 18 h of hypoxia followed by 3 h of reoxygenation. Quercetin (200 mg/kg body weight) administered to hypoxia challenged NY1DD mice in a single intraperitoneal (i.p.) dose at the onset of reoxygenation completely ameliorated all hypoxia reoxygenation (H/R)-induced pathophysiology. Additionally, it ameliorated the mild intrinsic steady state pathophysiology. These results are comparable with those seen with semisynthetic supra plasma expanders. In control mice, C57BL/6J, hypoxia reoxygenation-induced vaso-occlusion was at significantly lower levels than in NY1DD mice, reflecting the role of sickle hemoglobin (HbS) in inducing vaso-occlusion; however, the therapeutic benefits from quercetin were significantly muted. We suggest that these findings represent a unique genotype of the NY1DD mice, i.e., the presence of high oxygen affinity red blood cells (RBCs) with chimeric HbS, composed of mouse α-chain and human β^S^-chain, as well as human α-chain and mouse β-chain (besides HbS). The anti-anemia therapeutic benefits from high oxygen affinity RBCs in these mice exert disease severity modifications that synergize with the therapeutic benefits of quercetin. Combining the therapeutic benefits of high oxygen affinity RBCs generated in situ by chemical or genetic manipulation with the therapeutic benefits of antiadhesive therapies is a novel approach to treat sickle cell patients with severe pathophysiology.

## 1. Introduction

The vaso-occlusive crisis (VOC) is a common (yet challenging) complication in patients with sickle cell disease (SCD). It generally requires admitting the patients to the hospital. VOC episodes are induced by multifactorial processes that involve abnormal blood rheology, ischemic inflammation, increased oxidative stress, vascular adhesion, coagulation, and vascular tone modulation [1]. However, the basal level of vaso-occlusion is a normal feature of SCD, and the crisis is generally induced in patients by stressful events. Recent studies have shown that oxidative phenomena play significant roles in the severity of the pathophysiology of SCD [2,3,4]. Oxidative stress may even contribute to the sickling process through the formation of dense cells, the development of vaso-occlusive leukocytes, and shortened red blood cell (RBC) survival. The oxidant damage in sickle red blood cells (sRBCs) is most likely a consequence of the inherent increased autoxidation of oxy sickle hemoglobin [5,6], which results in a concomitant increase in free radical generation [7] with long-term effects of impairing the intrinsic antioxidant defense mechanism [8,9,10]. The sustained intracellular production of free radicals—coupled with polymerization—results in damage to the RBC membrane, and contributes to both hemolysis and vaso-occlusion. Hemolysis increases the concentration of free hemoglobin (Hb) in circulation, further contributing to vaso-constrictive events, which further enhance oxidative stress, the Fenton reaction [11], and activation of the endothelium and leukocytes.

The inflammatory responses seen in SCD patients are caused by ischemia reperfusion injuries that end in endothelial dysfunction and concomitant reduction in the NO production through shear thinning of the blood. Endothelial dysfunction is an important hallmark of SCD pathophysiology. In vivo and in vitro studies have demonstrated the susceptibility of vascular endothelial cells to the detrimental effects of ischemic reperfusion injury. Previous reports have established that post-capillary venular responses to ischemia reperfusion include abundant leukocyte adhesion, emigration, and enhanced oxidant production in transgenic sickle mice [12].

There have been advancements in several therapeutic strategies in recent years, to attenuate oxidation-mediated damage in SCD. The recent U.S. Food and Drug Administration (FDA) approval of the drug Endari (L-glutamine oral powder, Emmaus Medical, Inc., Torrance, CA, USA) for treating SCD patients reflects the potential benefits of antioxidant therapy as a disease modifying approach [13]. There is increased interest in exploring the therapeutic potential of natural products, general use antioxidants, and nutritional supplements, which may have fewer side effects than synthetic antioxidant drugs. Flavonoids are polyphenolic molecules that are plant metabolites and provide health benefits through cell signaling pathways (mTOR signaling, mitogen-activated protein kinases, protein kinase C and NF-κB signaling etc.). It was proposed that phenolic phytochemicals exert positive health effects in chronic disease states, including cancer [14], neurodegenerative disorders [15], and cardiovascular diseases [16]. Quercetin (3,3′, 4′, 5, 7-pentahydroxy flavone) is a member of the flavonoid family, natural antioxidants that exert their effects by inhibiting lipid peroxidation through blockades of the enzyme xanthine oxidase, chelating iron and directly scavenging hydroxyl [17] and superoxide anion radicals [18]. Quercetin was also reported to have potent anti-inflammatory, anti-proliferative, and anti-atherosclerotic activity [19]. Quercetin, in a dose-dependent manner, significantly improved arterial blood pressure, peripheral vascular resistance, and partially protected blood glutathione levels [20].

Many transgenic mice models of SCD were developed over the years with different degrees of disease severity. These studies have shown that replacing mouse β-chain with human β^S^ chain is not adequate to induce severe pathophysiology of SCD in mice. Both mouse α- and mouse β-chains need to be replaced with human α- and human β^S^-chains, respectively, to induce severe sickle cell pathophysiology, as seen in the transgenic BERK sickle mouse model. Murine transgenic models of SCD, such as the NY1DD [21], with around 50% of mouse α-chains, do not exhibit severe SCD-style pathophysiology reflected by vaso-occlusion or by anemia. In an S + S Antilles mouse, even partial replacement of the β^S^-chains with β^S^-Antilles-chains, which have a higher polymerization potential than β^S^-chains, does not induce severe sickle cell pathophysiology. These mice have no anemia, with hematocrit very close to that in wild type, and with small amounts of vaso-occlusion and mild levels of impairment of blood flow. The mild pathophysiology of disease in these transgenic mice, with a significant level of mouse α-chain and a small amount of mouse β-chain, was suggested to be a consequence of the β^S^-chain dependent polymerization inhibitory activity of mouse α-chain [22]. Though the polymerization inhibitory activity of mouse α-chain has been confirmed, inhibitory activity is significantly lower than the ϒ-chain of the fetal Hb. This suggests that other factors may also be contributing to the mild pathophysiology of these models. One possibility is the higher oxygen affinity of mouse α-chain and human β^S^-chain chimeric HbS and human α-chain and mouse β-chain chimeric Hb functioning as anti-anemia components, accomplishing targeted oxygenation of hypoxic areas in the body. The contribution to the mild pathophysiology of these chimeric mice has not attracted much attention; instead, attention is focused on the polymerization inhibitory activity of the mouse α-chain.

In the present study, we explored the potential therapeutic benefits of quercetin during H/R-induced vaso-occlusive episodes using a NY1DD transgenic sickle mouse model, which expressed chimeric HbS and RBC with high oxygen affinity. We selected an 18 h hypoxia and 3 h reoxygenation window to induce vaso-occlusion in NY1DD mice to evaluate the efficacy of the antioxidant therapeutic activity of quercetin in the presence of chimeric high oxygen affinity Hb. Together, the higher oxygen affinity chimeric Hb with the antioxidant quercetin provide a potential synergetic benefit that has not been previously recognized.

## 2. Methods

### 2.1. Animal Model

Male and female C57BL/6J wild type control and transgenic sickle (NY1DD) mice weighing approximately 25–35 g (4–6 months old) were used in this present study. Male and female C57BL/6J mice were obtained from Jackson Laboratories and were bred in the animal facilities at Albert Einstein College of Medicine. NY1DD mice were backcrossed, an average of 8 generations, into the C57BL/6J background. In NY1DD mice, β^S^-globin forms symmetrical tetramers with human α-globin (42%) and with mouse α-globin (~30%). In these mice, the total β^S^-globin levels were ~75% β^S^ of all β-globins [21]. For the present study, we selected NY1DD mice based on their highly exaggerated responses to hypoxia reoxygenation (H/R) compared with C57BL/6J mice, as reported earlier [12,21]. NY1DD mice show mild pathology but exhibited multiple organ damage and no hemolysis under normoxia. Under H/R, these mice demonstrated increased microvascular vaso-occlusion; therefore, they were used to simulate VOC in SCD. The systemic hematocrit (%) in NY1DD mice is like that found for C57BL/6J control mice (mean ± SD: NY1DD mice, 47.5 ± 3; controls, 46.5 ± 2.1) [23]. These mice were maintained on a standard diet and water, ad libitum. All experimental procedures were approved by the Einstein Animal Care and Use Committee and were consistent with PHS recommendations.

### 2.2. Preparation of Quercetin

Quercetin was obtained from the AMI Life Sciences Pvt. Ltd., Vadodara, and Gujarat, India. Stock solution of quercetin was prepared by the method as described earlier [24]. The quercetin was first dissolved in 50% dimethyl sulfoxide (DMSO) and then diluted to a final DMSO concentration of 2%. At this concentration, DMSO had no appreciable effect on Hb oxidation, Hb binding to RBC membranes, and lipid peroxidation [24].

### 2.3. Experimental Design

**Figure 1:**
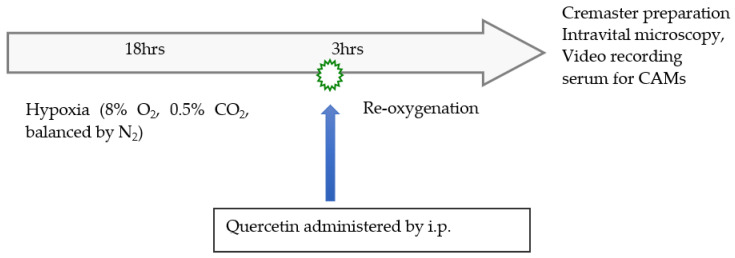
Schematic representation of experimental design.

Schematic representation of experimental design was presented in Figure 1. The experimental protocol consists of the following groups. Group 1: normoxia wild type mice control (C57BL/6J). Group 2: normoxia NY1DD mice control. Group 3: NY1DD mice were subjected to 18 h hypoxia (8% O_2_, 0.5% CO_2_, balanced by N_2_ in an Oxymax hypoxia chamber) followed by 3 h reoxygenation at ambient air. Group 4: NY1DD mice were subjected to the same 18 h hypoxia, but immediately after hypoxia, the animals were taken out from the hypoxia chamber, and quercetin (200 mg/kg) was administrated intraperitoneally (i.p.). After quercetin treatment, mice were exposed to 3 h of reoxygenation at ambient air, as described [25]. In groups 1, 2, and 3, mice were injected via i.p. with mouse phosphate buffered saline (1XPBS). Each group contained four mice.

### 2.4. Intravital Microscopy Studies

At the end of the reoxygenation period, mice were anesthetized with 10% urethane and 2% α-chloralose in saline (5 mL/kg) i.p. The animals were tracheostomized and in vivo microcirculatory observations were made in the open cremaster muscle postcapillary venules [26]. The suffusion and maintenance of the mouse cremaster preparation was conducted as described [27]. Intravital measurements were initiated after 15 min of the surgical exteriorization of the cremaster muscle and completed within the following 45 min. Microscopic observations were carried out using a Nikon microscope (model E400; Morrell Instrument Company, Inc., Melville, NY, USA) equipped with a Dage-MTI CCD television camera (model CCD-300T-RC, Dage-MTI Inc., Michigan City, IN, USA), and recorded by a Sony U-matic video recorder (model VO5800; Sony, Teaneck, NJ, USA). Micro-hemodynamic parameters were determined in randomly chosen postcapillary venules. Vessel luminal diameter (D, ~μm) was measured online using an image-shearing device (model 907, Instruments for Physiology and Medicine, San Diego, CA, USA). Red blood cell velocity (Vrbc) was measured along the vessel centerline using the ‘dual-slit’ photodiode and a velocity cross-correlator (model 102 BF, Instruments for Physiology and Medicine, San Diego, CA, USA), as described [28,29]. The centerline Vrbc was converted to the mean Vrbc across the vessel diameter using a conversion factor of 1.6 (Vrbc/Vmean = 1.6) as described [30]. Volumetric flow rates (Q) were determined from Vmean and the vessel cross-sectional area (πD2/4), as described [30,31]. The wall shear rate along the wall of the microvessel of a given luminal diameter (D) was calculated using the relationship = 8 Vmean/D [31]. Rolling leukocyte flux (cells/min) was determined as the number of leukocytes rolling through a given point in a vessel. Adherent leukocytes were counted along the length of a given venules and expressed as the average number of cells per 100 μm length of the vessel. A leukocyte was considered as adherent if it remained stationary for longer than 30 s. Trans-endothelial emigration (extravasation) of leukocytes were determined on-line, as the number of interstitial leukocytes in the field of view adjacent (within 30 μm) to venules. 

### 2.5. Estimation of Lipid Peroxidation Product in Plasma

Lipid peroxidation levels in plasma were estimated by measuring thiobarbituric acid reactive substances (TBARS) and expressed in terms of malondialdehyde (MDA) content, which is the product of lipid peroxidation by using the OxiSelect^TM^ TBARS assay kit (Cell Biolabs Inc., San Diego, CA, USA). 

### 2.6. Assay of Circulating Plasma Levels of Cell Adhesion Molecules (CAM)

Soluble E-selectin, sP-selectin, and sVCAM-1 were quantified from the plasma by the standard ELISA method using a Quantikine kit (R&D Systems, Minneapolis, MN, USA).

### 2.7. Statistical Analysis

Statistical analysis was performed with GraphPad Prism-6 software (GraphPad Software, Inc., La Jolla, CA, USA). Kruskal–Wallis and Mann–Whitney U-tests were used for comparisons between groups. The results are expressed as means ± standard error of the mean (SEM). *p* values less than 0.05 was considered significant. 

## 3. Results

### 3.1. Selection of Quercetin Dose

Although the nutraceutical use of quercetin is primarily through oral administration—in the present study, we administered quercetin to wild type mice and SCD mice by the i.p. route to assess the therapeutic benefits quickly. A pilot study involving three groups, with four mice per group (50, 100, and 200 mg/kg body weight) was performed to characterize the dose-dependent effect of quercetin after a single i.p. dose. All concentrations used in this study induced a quercetin therapeutic benefit, but the effect exerted by 200 mg/kg quercetin provided the most significant improvement in vaso-occlusive and hemodynamic parameters in postcapillary venules, and was thus chosen as the dose for the present study. 

### 3.2. Qualitative Video Microscopic Evaluation of SCD Therapeutic Activity of Quercetin

**Figure 2:**
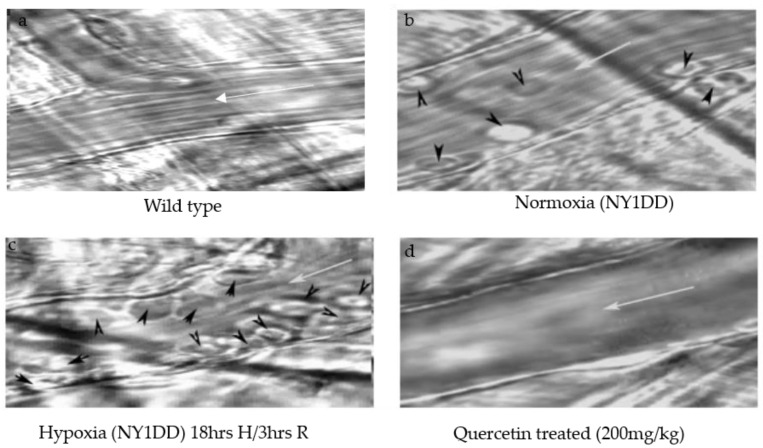
Intravital microscopy of cremaster venules of transgenic sickle cell mice (NY1DD) showing ameliorating effects of single dose quercetin (200 mg/kg, i.p.) on leukocyte adhesions and extravasation. (**a**) Wild type control mice; (**b**) normoxic NY1DD mouse shows few rolling leukocytes and adhesion; (**c**) after 18 h of hypoxia and 3 h of reoxygenation, cremaster venules show marked leukocyte adhesion, as well as emigrated leukocytes (arrows head); (**d**) administration of quercetin at the onset of reoxygenation in NY1DD mice almost completely abolished leukocyte adhesion and emigration. White arrow (→) indicates the blood flow direction and black arrowhead indicates leukocytes.

A representative video microscopic image of cremaster postcapillary venules of NY1DD before and after H/R, and the influence of the quercetin treatment after hypoxia, are shown in Figure 2, and compared with wild type mice (Figure 2a). In NY1DD mice under normoxic conditions (Figure 2b), we observed intrinsic mild pathology symptoms by means of leukocyte adhesion and emigration in postcapillary venules. However, after H/R, leukocyte adhesion, and emigration increased (Figure 2c) significantly compared to the control wild type mice (Figure 2a). After quercetin treatment, vaso-occlusion clearly resolved, as seen in Figure 2d, suggesting that quercetin completely attenuates the adherent leukocytes and emigration.

### 3.3. Influence on Vaso-Occlusion

In attempt to further investigate the effects of quercetin on vaso-occlusion, we studied the venular diameter, leukocyte rolling flux, adhesion, and emigration in postcapillary venules; these results are shown in Table 1. Leukocyte rolling and capturing are associated with the initial steps of inflammatory response. We observed a significant (*p* < 0.0001) increase in the leukocyte rolling flux in untreated NY1DD mice subjected to H/R compared to the normoxia NY1DD mice and wild type control. Quercetin treatment at the onset of reoxygenation reduces the number of leukocyte rolling flux in cremaster postcapillary venules. It was reported earlier that NY1DD sickle mice exhibit intrinsic, very low levels of vaso-occlusion and impaired blood flow, which increases during H/R [12]. In a steady state, NY1DD mice experience an increase in leukocyte adhesion (*p* < 0.028) in postcapillary venules as compared to wild type mice. Hypoxia/reoxygenation in NY1DD mice further increases this inflammatory response as evidenced by a ~3-fold increase in leukocyte adhesion when compared to normoxic NY1DD mice (*p* < 0.0001). We also observed a significant (*p* < 0.0001) reduction in leukocyte adhesion after treating with quercetin (200 mg/kg) at the onset of reoxygenation as compared to untreated NY1DD mice, and did not show any significant difference compared to wild type mice. The difference in the venular diameter after quercetin administration is marginal when compared to the untreated hypoxic sickle mice.

In the NY1DD mouse, inflammatory response to H/R was evident by leukocyte extravasation [12] that reflected the leakiness of the endothelium. Even under steady state conditions, NY1DD mice exhibit ~7-fold increased leukocyte emigration as compared to wild type mice (*p* < 0.028). NY1DD mice subjected to H/R exhibited a markedly increased inflammatory response as evidenced by a significant (*p* < 0.0001) increase in leukocyte extravasation in postcapillary venules. Treatment with quercetin (200 mg/kg) at the onset of reoxygenation resulted in pronounced reduction in emigrated leukocytes when compared to untreated NY1DD mice subjected to H/R (*p* < 0.0001). Leukocyte emigration in the treated group was significantly (*p* < 0.019) different from normoxic NY1DD mice that had not undergone H/R. Vaso-occlusion, both intrinsic (steady state) and induced by hypoxia in NY1DD mice, were normalized, nearly to wild type level, after quercetin treatment.

### 3.4. Influence on Microcirculation

#### 3.4.1. Effect of Quercetin on Vrbc in Post-Capillary Venules

**Figure 3:**
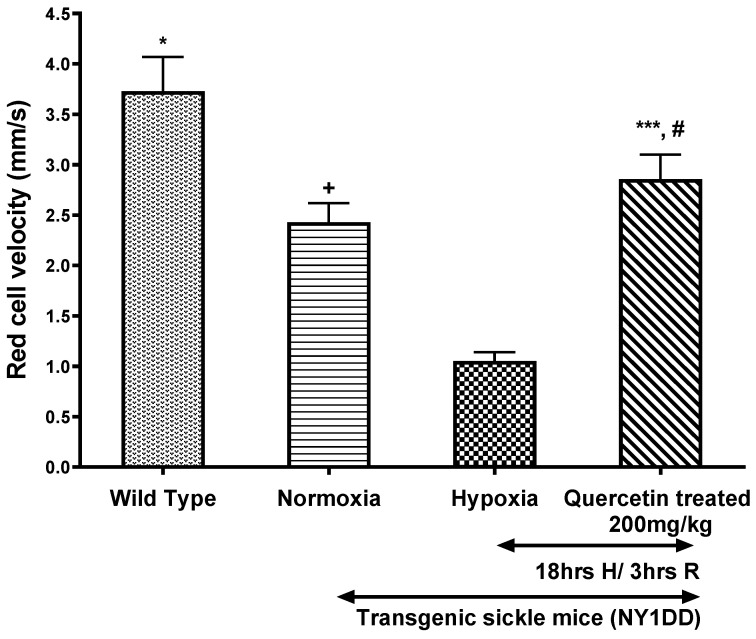
Effect of single dose of quercetin (200 mg/kg, i.p.) on red cell velocity (Vrbc) in wild type and NY1DD transgenic sickle mice under normoxia and hypoxia conditions. Values represent the mean ± SEM for four animals in each group; ^+^ *p* < 0.021 as compared to normoxic wild type controls; * *p* < 0.0001 as compared to H/R-induced NY1DD mice; ^#^ not significant as compared to normoxic NY1DD mice; *** *p* < 0.0001 as compared to NY1DD mice subjected to H/R (untreated).

We observed a significant decrease in Vrbc in the postcapillary venules of NY1DD mice subjected to H/R as compared to wild type. Prior treatment with quercetin (200 mg/kg) at the onset of reoxygenation significantly (*p* < 0.0001) attenuates the hypoxia-induced decrease in Vrbc compared to untreated NY1DD mice subjected to H/R (Figure 3).

#### 3.4.2. Effect of Quercetin on Wall Shear Rate and Q in Post-Capillary Venules

**Figure 4:**
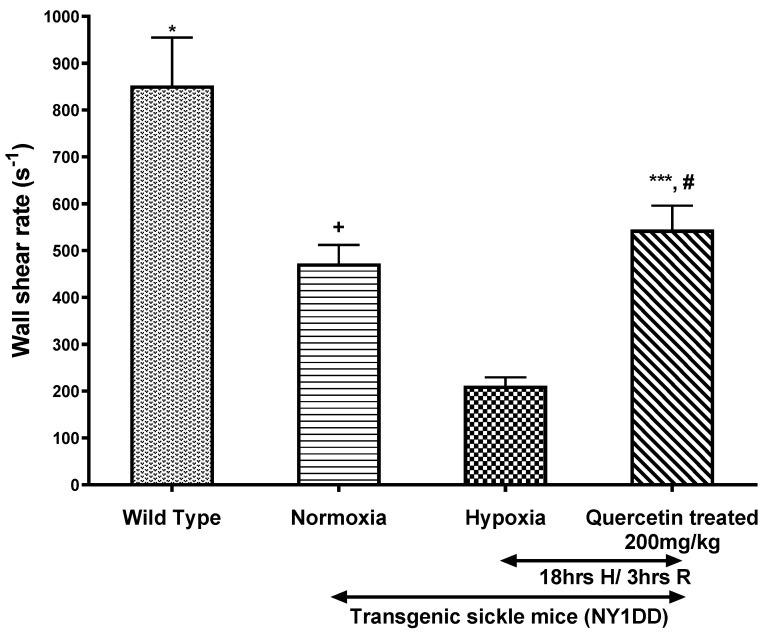
The effect of a single dose of quercetin (200 mg/kg, i.p.) on the wall shear rate in wild type and NY1DD transgenic sickle mice under normoxia and hypoxia conditions. Values represent the mean ± SEM for four animals in each group; ^+^ *p* < 0.03 vs. normoxic wild type controls; * *p* < 0.0001 as compared to H/R-induced NY1DD mice; ^#^ non-significant vs. normoxic NY1DD mice; *** *p* < 0.0001 vs. NY1DD mice subjected to H/R (untreated).

**Figure 5:**
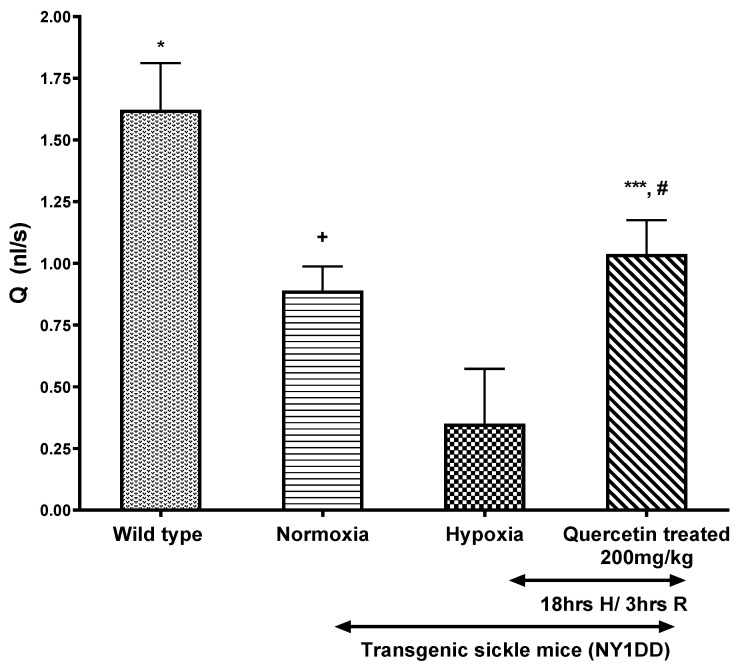
The effect of a single dose of quercetin (200 mg/kg, i.p.) on volumetric flow rate (Q) in wild type and NY1DD transgenic sickle mice under normoxia and hypoxia conditions. Values represent the mean ± SEM for four animals in each group; ^+^ *p* < 0.02 vs. normoxic wild type controls; * *p* < 0.0001 as compared to H/R-induced NY1DD mice; ^#^ non- significant vs. normoxic NY1DD mice; *** *p* < 0.0001 vs. NY1DD mice subjected to H/R (untreated).

The effect of quercetin on micro-vascular flow parameters, such as wall shear rate and Q, are presented in Figure 4 and Figure 5, respectively. H/R caused ~5-fold decline in wall shear rates and Q in NY1DD mice when compared to control wild type mice. In contrast, treatment with quercetin, after the onset of reoxygenation in NY1DD mice, resulted in a marked ~3-fold increase in the wall shear rate and Q when compared to the untreated hypoxic NY1DD mice. However, these treated mice were not significantly different from normoxic NY1DD mice. All hemodynamic parameters (Vrbc, wall shear rate, and Q) significantly increased after treatment with quercetin vs. untreated NY1DD mice exposed to H/R. However, those parameters did not fully revert to wild type control mice.

### 3.5. Influence on Biomarkers

#### 3.5.1. Effect of Quercetin on Lipid Peroxidation

**Figure 6:**
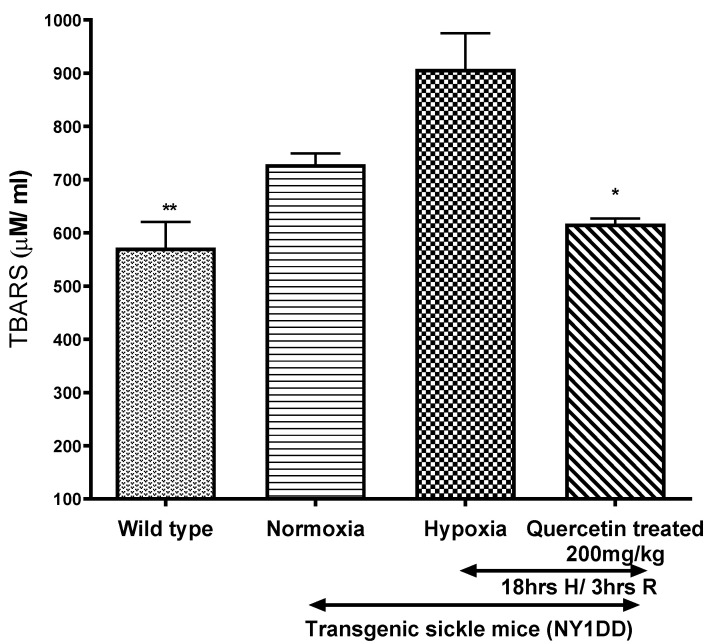
Effect of single dose of quercetin (200 mg/kg, i.p.) on thiobarbituric acid reactive substances (TBARS) in wild type and NY1DD transgenic sickle mice under normoxia and hypoxia conditions. Values represent the mean ± SEM for four animals in each group; ** *p* < 0.01 vs. hypoxia NY1DD mice; * *p* < 0.028 as compared to H/R-induced NY1DD mice.

Oxidative stress in NY1DD sickle mice were measured in plasma by using the TBARS assay kit (Figure 6). During normoxia, plasma level of TBARS were higher in NY1DD mice than wild type control mice. TBARS increased significantly (*p* < 0.02) in NY1DD after exposure to H/R. Quercetin treatment reduced plasma TBARS significantly (*p* < 0.02) in NY1DD mice subjected to H/R compared to untreated NY1DD mice, to nearly that observed in wild type control mice. 

#### 3.5.2. Effect of Quercetin on Soluble Cell Adhesion Molecules (CAM)

**Figure 7:**
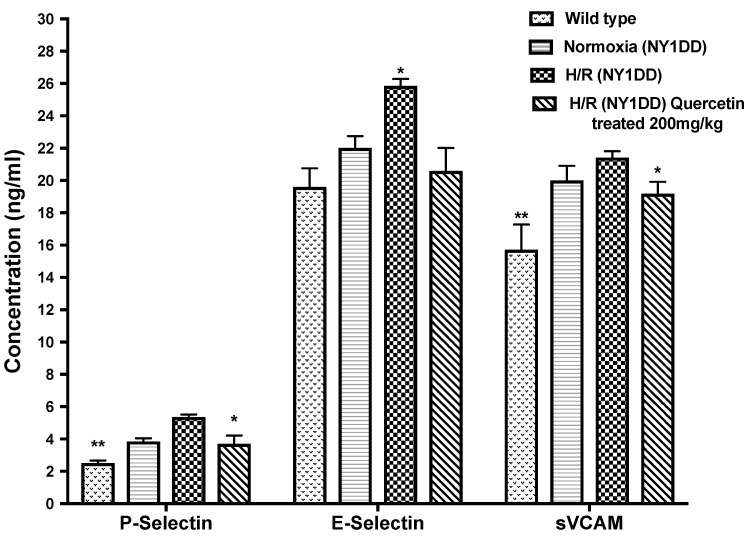
Effect of single dose of quercetin (200 mg/kg i.p.) on endothelial activation markers, expression of soluble cell adhesion molecule, sVCAM-1, and selectins, sP-selectin, and sE-selectin in wild type and NY1DD transgenic sickle mice under normoxia and hypoxia conditions. Values represent the mean ± SEM for four animals in each group; P-selectin: ** *p* < 0.01 vs. hypoxia NY1DD mice; * *p* < 0.02 as compared to H/R-induced NY1DD mice. E –selectin: * *p* < 0.03 as compared to quercetin treated (200 mg/kg) H/R-induced NY1DD mice and wild type control mice. sVCAM: * *p* < 0.01 as compared to H/R-induced NY1DD mice. ** *p* < 0.007 vs. NY1DD mice subjected to hypoxia–reoxygenation (untreated).

We did not observe any significant difference in the CAM level between NY1DD and wild type control mice in steady state condition. Significant (*p* < 0.05) increases in the concentration of CAM (sP-selectin, sE-selectin, and sVCAM-1) were observed in NY1DD subjected to H/R compared to normoxia in NY1DD mice (Figure 7). Treatment with quercetin at the onset of reoxygenation of NY1DD subjected to hypoxia reduced significantly (*p* < 0.03) the concentration of sP-selectin, sE-selectin, and sVCAM-1 when compared to untreated NY1DD mice. 

### 3.6. Influence on Red Cell Density Pattern

**Figure 8:**
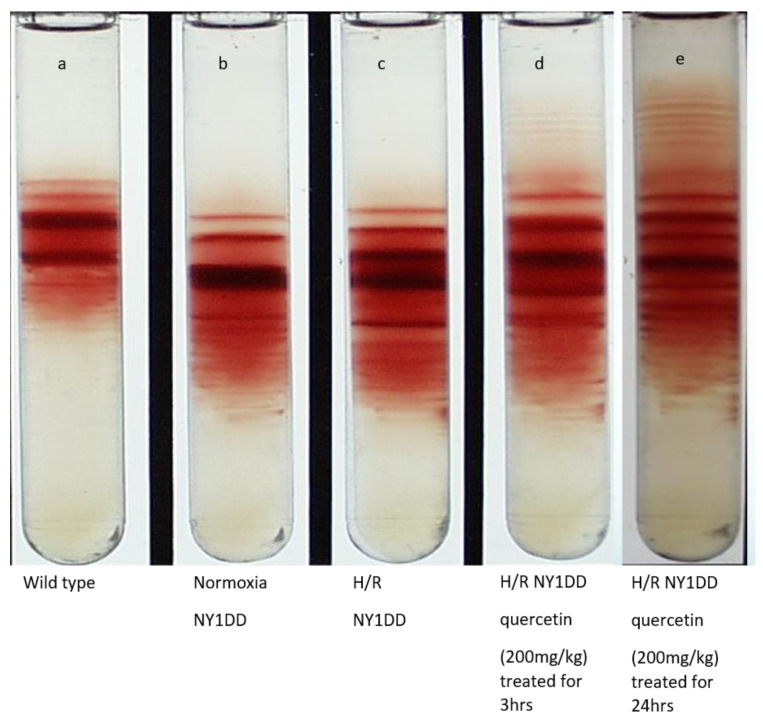
Effect of quercetin on red cell density pattern of wild type and NY1DD under normoxia and hypoxia condition; (**a**) wild type control mice; (**b**) normoxia NY1DD mice; (**c**) H/R-induced NY1DD mice; (**d**) H/R-induced NY1DD mice treated with quercetin (200 mg/kg) for 3 h; (**e**) H/R-induced NY1DD mice treated with quercetin (200 mg/kg) for 24 h.

Red cell density pattern of wild type and NY1DD mice are represented in Figure 8. The data clearly confirm that the pathophysiology of NY1DD mice (Figure 8b) is mild, and its pattern is very distinct from wild type (Figure 8a), reflecting the disease induced by the high density of RBC. This represents the dehydration of RBC in vivo in sickle mice. The H/R impacted the density patterns; there does not appear to be as remarkable a difference between the wild type and steady state level patterns in NY1DD mice. Treatment with quercetin for 3 h (Figure 8d) not only reversed the pattern, but it was comparable to the pattern seen in wild type mice. The pattern observed with treatment of quercetin after 24 h (Figure 8e) is better than 3 h treatment, suggesting quercetin can reverse the steady state pattern to normal levels. The reversal by quercetin induces a degree of regeneration of low-density RBC, which are presumably the RBCs that were damaged previously. The surprising aspect of therapeutic efficacy of quercetin is not limited to reversing the H/R-induced severity of the disease, but the regeneration of the overall patterns in NY1DD mice to a state better than the steady state with which we started.

### 3.7. The Efficacy of H/R to Induce Vaso-Occlusion in Control C57BL/6J Mice and Therapeutic Efficacy of Quercetin to Ameliorate the Induced Vaso-Occlusion

**Figure 9:**
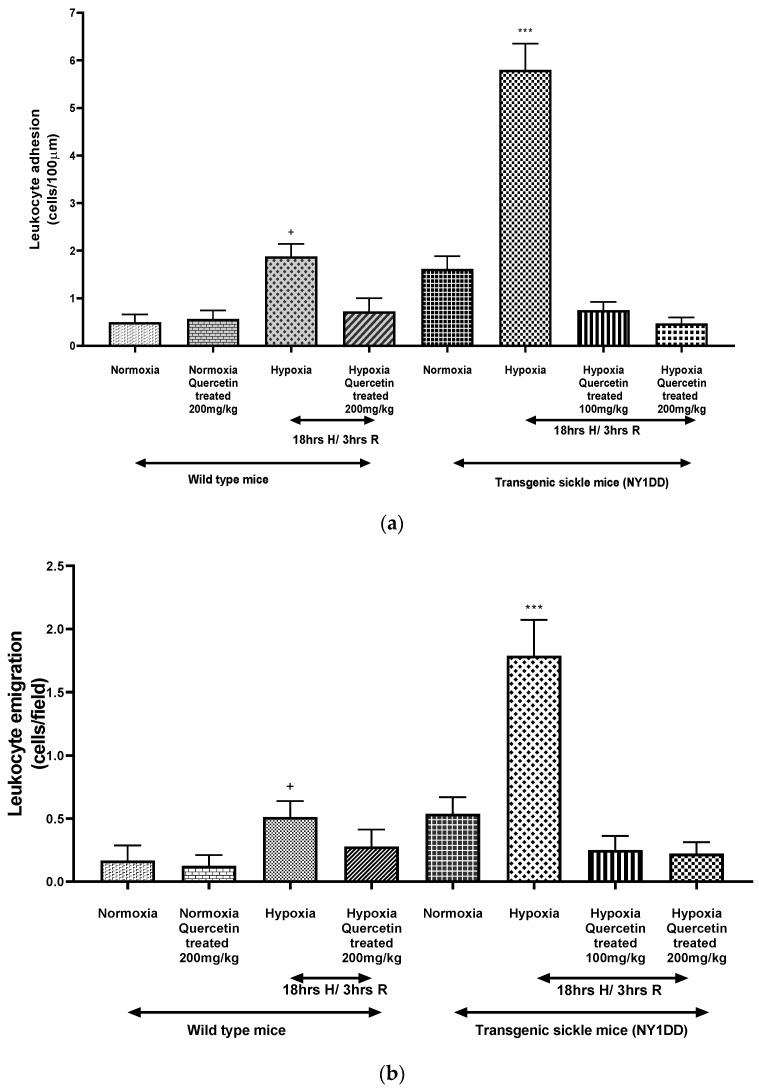
Comparison of the ability of H/R to induce vaso-occlusion in wild type mice vs. NY1DD mice and efficacy of quercetin to quench the H/R induced vaso-occlusive events. (**a**) Leukocyte adhesion; (**b**) leukocyte emigration. Values represent the mean ± SEM for four animals in each group; *** *p* < 0.0001 as compared to H/R-induced NY1DD mice treated with quercetin (100 and 200 mg/kg) and normoxic NY1DD mice; ^+^ *p* < 0.021 vs. normoxic wild type control.

The high efficacy of quercetin ameliorating vaso-occlusion in NY1DD mice and the regenerating pattern comparable to that of wild type mice is very surprising. It is indeed very unusual when compared to other antioxidant therapy studies carried out with sickle cell patients. Accordingly, we compared H/R to induce vaso-occlusion in wild type control mice and NY1DD mice; therapeutic efficacy of quercetin to ameliorate the vaso-occlusions are shown in Figure 9. We observed that wild type control mice H/R induced only very limited amounts of vaso-occlusion, noticeably lower than that induced in NY1DD (Figure 9a). This confirms that H/R-mediated induction of vaso-occlusion and accompanying severe sickle cell pathophysiology in NY1DD is a direct consequence of the presence of β^S^-chain dependent polymerization of HbS during hypoxia. The mild pathophysiology of disease in NY1DD in the steady state was attributed to the presence of mouse α-chain to a nearly 50% level in NY1DD mice. This is true in S + S Antilles mice as well. The complete replacement of the mouse α-chain and mouse β-chain with human α-chain and β^S^-chain can only induce severe SCD pathophysiology in transgenic mice as seen in BERK mice. 

The efficacy of quercetin to ameliorate the H/R induced vaso-occlusion in wild type control mice was only partial, despite the fact that the level of vaso-occlusion was considerably lower, i.e., high therapeutic efficacy of quercetin is unique to transgenic mice NY1DD but not unique to quercetin itself. Apparently, the presence of mouse α-chain in NY1DD and/or of small amounts of mouse β-chain induces a higher quercetin therapeutic efficacy in ameliorating the vaso-occlusion in NY1DD. The higher oxygen affinities of chimeric HbS in NY1DD composed of mouse α-chain and human β^S^-chain, or composed of human α-chain and mouse β-chain, could contribute to the unusual therapeutic activity of quercetin. 

## 4. Discussion

In NY1DD sickle mouse, H/R induces RBC sickling, leading to vaso-occlusion of RBCs that occlude the microvascular bed and induce ischemic tissue injury. H/R has been a useful tool in the study of red cell sickling, vaso-occlusion, and generation of reactive oxygen species (ROS) in SCD leading to cellular dysfunction and severe pathophysiology. Previous studies have reported that H/R promotes inflammation in transgenic mouse models of SCD and stimulates vascular endothelial cells to release extracellular superoxide radicals. Compared to the effect of H/R in wild type mice, the NY1DD transgenic sickle mouse H/R induced significantly worse vaso-occlusive episodes and an inflammatory response when compared to either the wild type control or the steady state ‘mild pathophysiology’ of the NY1DD [12]. In the present study, 18 h of hypoxia followed by 3 h reoxygenation resulted in severe microvascular vaso-occlusion, leukocyte rolling flux, emigration, and adhesion, lowering of hemodynamic parameters including Vrbc, and wall shear rate and flow (Q) in the NY1DD. The mild (pre-H/R) pathophysiology and potentially H/R induced amplified pathophysiology permitted us to evaluate the potential therapeutic activity of new therapeutic agents, including quercetin, to modify the severity of SCD. Specifically, the pathophysiology of SCD is reflected by a level of increase in vaso-occlusion, poor hemodynamic parameters, the biomarkers of oxidative/inflammatory stress, and density gradient pattern of RBC. In this study, we reported that quercetin was able to reverse each of these parameters, essentially reversing the H/R induced changes. In steady state, the mild pathophysiology of the NY1DD transgenic mouse has been attributed to the presence of the murine α-chain, which has an intrinsic anti-sickling activity in vivo [32]. However, the anti-sickling activity of the mouse α-chain is very small and considerably lower than that of the γ-chain. Additionally, the NY1DD chimeric hemoglobin S consisting of the mouse α-chain and human β^S^-chain [33] has a higher oxygen affinity relative to HbS. The mouse α-chain in the NY1DD mouse represents nearly 50% of the non-β chain, with the other 50% being human α-chains. The large amount of high oxygen affinity chimeric HbS can improve microcirculation and provide better oxygen delivery to the hypoxic tissue regions, additionally increasing the delay time to sickling [34], thereby modifying the severity of disease in the NY1DD sickle model. NY1DD also has small amounts of mouse β-chains, chimeras of these with human α also exhibit higher oxygen affinity, further contributing to the therapeutic activity, i.e., in modifying the β^S^-chain induced disease severity.

Treatment with quercetin, at the onset of reoxygenation in NY1DD mice, improved all parameters studied here. The attenuation of the vaso-occlusion reflected by the decrease in the number of leukocytes adhered in the postcapillary venules was near to wild type control mouse. Further, leukocyte rolling and emigration in the postcapillary venules were eliminated. The observed therapeutic effect might be from the ability of quercetin to scavenge the free radicals generated during H/R and the ongoing autoxidation of HbS. In vitro studies have shown that treatment of erythrocytes from sickle cell anemia patients with quercetin provide protection against hemoglobin oxidation and other cellular modifications promoted by peroxides [24]. In our study, quercetin protected the endothelium and RBC against damage from H/R in the absence of severe anemia. The complete reversal of vaso-occlusive events after 3 h reoxygenation in the NY1DD mice suggest that the use of quercetin as a therapeutic to modulate/moderate the severity of the disease is very promising. Further, it may be advantageous to use quercetin as a nutritional supplement with sickle pediatric populations to slow the rate of increasing oxidative damage, disease severity, and avoid/slow age-related decrease in the hematocrit. 

Quercetin treatment also reverses the impairment of all hemodynamic parameters induced by hypoxia; however, this reversal is not to the level of wild type control mice as seen in vaso-occlusion parameters. This suggests that quercetin attenuates all hemodynamic parameters generated by the hypoxia, but not the intrinsic disease severity in this transgenic NY1DD mice model. Earlier studies [35] have reported that hemodynamics depends on vessel diameter, pulse pressure, or changes in rheological parameters, i.e., RBC deformability (shear thinning of blood and endothelial NO production), RBC aggregation, RBC adhesion, hematocrit, and blood viscosity. The observed reversibility of hemodynamic parameters after treatment with quercetin suggest that the hypoxia-induced polymerization of HbS in RBC does not end up in generating irreversibly sickled RBC. Though plasma of SCD patients has been shown to contain free ferrous hemoglobin and its scavenging of increased quantities of NO [36,37], NY1DD mice are essentially free of hemolysis and the hypoxia-induced impairment of hemodynamics is mainly from ischemic reperfusion injuries.

Erythrocytes are not only the main source of ROS in SCD, but they sustain the brunt of intracellular oxidative stress. Normal RBCs are resistant to oxidative damage through their antioxidant system, particularly glutathione (GSH) and enzymes involved for the glutathione redox recycle. In sRBC, a very low concentration of GSH was reported and it was found that the loss of intracellular GSH possibly diminishes the antioxidant defense of RBCs, rendering them more susceptible to oxidative damage [38]. Lipid peroxidation is one of the main manifestations of oxidative damage initiated by ROS and has been linked to the altered membrane structure and enzyme inactivation [39]. Increased levels of lipid peroxidation in NY1DD mice subjected to H/R are reflected by the elevated levels of plasma TBARS in untreated hypoxic transgenic sickle mice, implicating an increased production of free radicals generated during H/R protocol. These free radicals initiate activation of the leukocytes and destruction of lipid membrane in RBC, which will eventually cause chronic hemolysis. Studies have shown that lipid peroxidation and OH-radical generation occur in the sickle mouse model under the same H/R experimental conditions [40]. An earlier study [41] also reported that increases in the levels of TBARS in the transgenic sickle mice (NY1DD) and in transgenic sickle knockout (BERK) mice were accompanied by marked decreases in antioxidant enzymes (i.e., total SOD, catalase, and glutathione peroxidase) in the tissues compared with control C57BL/6J mice.

In a previous study, [42] reported that high oxygen affinity generated in situ using 5-hydroxymethyl furfural (5-HMF) was shown to provide protection against hypoxia, particularly increasing the oxygen saturation in blood during hypoxia. Recently, 5-HMF was suggested as therapeutic in attenuating the hypoxemia effect in COVID patients [43]. Accordingly, it is conceivable that the NY1DD transgenic RBC may be capable of exerting an anti-anemia or anti-ischemia effect and play a role in modifying the severity of both the steady state disease and H/R pathology. There may also be an intrinsic contribution from the β^S^-chain dependent polymerization inhibitory activity of mouse α-chains. Previously, we had not considered the potential disease modifying effects of high oxygen affinity of chimeric HbS in this model or other transgenic sickle models.

The severity of the disease in NY1DD mice significantly increased by H/R, as seen by many parameters, including an increase in vaso-occlusion, the collapse of micro circulatory functioning, an increase oxidative stress as reflected by oxidative stress markers, and an increase in immune cell biomarkers and patterns of RBC by density gradient, which are comparable to the BERK mice model. However, the changes seen in the density gradient pattern of RBC, after 3 h of reoxygenation following an 18 h hypoxia, does not reflect a severity of the disease to the level seen in BERK mice in a steady state, as a severe model of SCD.

The observed high therapeutic efficacy of quercetin in NY1DD mice was also seen earlier with supra plasma expanders EAF P5K6 Alb Tempol 12 and EAF P3K6 Hb. The present study, demonstrating that quercetin reverses the mild intrinsic disease severity in NY1DD mice, is a unique observation, and we suggest that this high efficacy might be due to the antioxidant therapeutic activity of quercetin synergized with intrinsic anti-anemia therapeutic activity towards high oxygen affinity RBC present in the NY1DD mice model.

An increase in the biomarkers of oxidative stress/inflammatory stress observed in H/R-induced NY1DD mice is consistent with earlier studies. In steady state, the level of these biomarkers in NY1DD are not significantly different from that in wild type. However, quercetin reverses the increased level of all biomarkers. The present study demonstrates that treatment with quercetin normalizes the levels of TBARS increased by H/R-induced NY1DD mice. Several studies have reported that quercetin delays oxidative pathway mediated cell injury by scavenging free radicals, affording protection against lipid peroxidation and chelating metal ions [44,45,46]. The protective effect of quercetin appears to be operational in H/R induced oxidative stress in transgenic sickle mice.

Cell adhesion molecules (CAMs), such as sVCAM-1, E-selectin, and P-selectin, participate in leukocyte adhesion to the endothelium, and play an important role for inflammation. Overexpression of these CAMs is also a common feature in inflammation, induced by pro-inflammatory cytokines, growth factors, platelet activator, and chemotactic factors in the blood. In contrast, as part of the biochemical program that facilitates blood flow and maintains vascular homeostasis, NO normally suppresses expression of VCAM-1, ICAM-1, and E-selectin [47,48]. The impairment of NO bioavailability results in pathological activation of endothelial cells to express adhesion molecules. An increased level of sVCAM-1, sP-selectin, and sE-selectin was observed in untreated hypoxia transgenic sickle mice, indicating the impaired NO bioavailability, which further enhances the expression of cell adhesion molecules and causes vaso-occlusion. The data presented here are consistent with the reports by Belcher et al. [49] regarding the transgenic sickle mice model and SCD patients [50]. We observed the near normalization of levels of CAM in transgenic sickle mice after treatment with quercetin at the onset of reoxygenation. It was previously documented that flavonoids act as anti-inflammatory agents to inhibit the expression of CAMs, and the effects depend on their bioavailability [20,24]. It was previously reported that, in healthy volunteers who supplemented their diets with 50, 100, or 150 mg/d quercetin aglycone for 2 weeks, there was a significant, dose-dependent accumulation of quercetin in plasma. The pharmacokinetics of plasma quercetin from quercetin aglycone differed depending on the quercetin dosages given. The AUC as a measure of bioavailability ranged from 76.1 μmol·min·L^−1^ (50 mg dosage) to 305.8 μmol·min·L^−1^ (150 mg dosage) [51].

The density patterns of RBCs in the normoxic NY1DD suggest that these RBCs are denser than wild type control mice, which would reflect the mild pathophysiology of the disease state. However, noticeable changes were seen in the density patterns after H/R in NY1DD mice. Prior treatment with quercetin at two different time points, 3 and 24 h at the onset of reoxygenation, improves the RBC density gradient pattern near to that in the steady state condition.

We previously reported that the therapeutic efficacy of Tempol conjugated to albumin was marginal in sickle mice models [52]. However, when Tempol was conjugated to EAF P5K6 Alb, a supra plasma expander, the therapeutic effect seen was comparable to what we observed in the present study with quercetin. A very interesting finding is that a high efficacy of quercetin does not just ‘quench’ the H/R-induced vaso-occlusion, but returns the animal to steady state base line conditions. As noted above, we saw this effect earlier with supra plasma expanders as a novel class of therapeutics to treat SCD [52]. We now attribute this to the intrinsic ability of NY1DD mice to modify the disease induced by the transgene human β^S^-chain to a milder pathophysiology because of the higher oxygen affinity of chimeric HbS present in these mice and composed of mouse α-chain and human β^S^-chain [21]. The same chimeric HbS also facilitate the recovery from H/R-induced expression of severe pathophysiology of the disease. Therapeutic benefits of high oxygen affinity RBC improving the microcirculation, and better delivery of oxygen, were demonstrated by Marcos Intaglietta and his colleagues [53]. Accordingly, we suggest high therapeutic efficacy of quercetin to reverse the severe pathophysiology induced by H/R, and reverse the state comparable to wild type mice. The observed results essentially reflect a combination therapy–therapeutic activity of high oxygen affinity RBC, intrinsic to these mice models, coupled with antioxidant therapeutic activity of quercetin.

It should be noted that chimeric Hb in NY1DD mice models provides a significant modification of the pathophysiology of SCD induced by β-chain-dependent polymerization in a steady state, but chimeric HbS does not provide protection against H/R. Since the oxygen affinity of chimeric HbS is slightly higher than HbS, it apparently gives additional time for RBC with the HbS to pass through capillaries, improving the tissues oxygenation in the process. However, under hypoxic conditions, chimeric HbS polymerize just as HbS, thereby failing to provide protection from H/R. The wild type mice do not induce severe vaso-occlusion or pathologies, as seen with NY1DD, reflecting the need for RBC containing polymerized deoxy HbS to induce vaso-occlusion and other concomitant pathophysiologies. The oxygen affinity for chimeric HbS is slightly higher than HbS, whereas HbS modified by Voxelotor is expected to have an oxygen affinity around 2 mm Hg. Therefore, it is conceivable that Voxelotor could provide protection against H/R. It should be noted that EAF P3K6 Hb, a high oxygen affinity ‘nano oxygen pump’ that increases the oxygen extraction from RBC, as well as facilitates a better oxygenation of RBC in the lungs, when present during hypoxia, provides protection against H/R-induced changes in NY1DD.

We should also note that modifying the SCD severity by increasing the oxygen affinity of HbS in RBC in situ has been a major approach to treat SCD; the development Voxelotor by Global Blood Therapeutics represents the first such successful approach [54]. However, the scientific community has been suspicious of the potential benefit of this approach. It was argued that increasing the oxygen affinity of HbS will amplify the intrinsic anemia present in such patients [55]. On the other hand, Eaton and colleagues argued that increasing the oxygen affinity of HbS in RBC in situ could increase the delay time and provide sufficient time for the RBC to transit through the capillary/microcirculation, thereby increasing the efficacy of tissue oxygenation in these sickle cell patients, an anti-anemia therapeutic activity [56]. We believe that such a therapeutic effect is intrinsic to NY1DD mice, and therapeutic benefits seen in these models with test samples are likely to be reflecting a combination therapy. This thought process argues that combining high oxygen affinity inducing therapeutic approaches with antioxidant therapy may be very valuable tools to treat SCD.

The homozygous sickle disease outside the United States and the African continent is generally associated with mild pathophysiology. Sickle cell patients in mid-eastern countries and the Indian subcontinent are generally associated with an expression of fetal Hb, even as adults. The disease modifying therapeutic benefit of fetal Hb has been attributed to the deoxy HbS polymerization inhibitory activity of the ϒ-chain of fetal Hb [57]. However, recent studies on the therapeutic benefits of high oxygen affinity RBC, in terms of improving microcirculation and increasing the oxygen delivery, need to reconsider the potential contribution of fetal Hb as a high oxygen affinity Hb in RBC as well [58]. The contribution of increasing the peripheral oxygenation level in sickle cell patients was also recently recognized [59], and this is essentially another contribution of fetal Hb in eliciting an anti-anemia therapeutic activity. The contribution of EAF P5K6 Alb as therapeutic to modify the severity of SCD is through supra plasma expander activity, which facilitates improved tissue oxygenation, and can be considered an anti-anemia therapeutic benefit [52]. Combining it with Tempol, it develops a novel therapeutic, with SCD therapeutic activity as effective as EAF P3K6 Hb (an oxygen therapeutic) in attenuating the mild disease pathology of NY1DD mice. However, therapeutic benefits of EAF P5K6 Alb on the systemic circulation in BERK mice induces a degree of toxicity in cerebral circulation, does not normalize the increased CBF in BERK mice, and creates an oxygen debt. On the other hand, EAF P3K6 Hb, shows the therapeutic benefits in systemic circulation as well as cerebral circulation, and does not induce an oxygen debt in the brain. Accordingly, we suggest combination antioxidant therapy using quercetin with hydroxyurea therapy might induce the generation of high oxygen affinity fetal Hb, and can further modify the mild pathophysiology of disease in mid-eastern countries and the Indian subcontinent. Quercetin therapy could also be synergized with the newly emerging disease modifying efforts using high oxygen affinity RBC generation in situ.

In the present study, we used an i.p. route to administer quercetin to obtain a quick response of the therapeutic benefits. The oral administration of quercetin may be a better approach to provide therapeutic benefits in a steady state, and the infusion of quercetin should be considered with patients admitted to the hospital for vaso-occlusive crisis, to get faster relief from the crisis and shorten the hospital stay.

## 5. Conclusions

A single dose of quercetin (200 mg/kg) given to NY1DD sickle mice subjected to hypoxia at the onset of reoxygenation not only attenuates the hypoxia-induced pathophysiology, but it brings the parameters near to wild type control mice. This high efficacy of disease modifying activity of quercetin seen in the present study is comparable to the SCD therapeutic benefits of a new class of molecules, semisynthetic supra plasma expanders, studied by us earlier. We suggest that high therapeutic efficacy of quercetin in NY1DD mice reflects the genotype of this mouse model, namely the presence of high oxygen affinity chimeric HbS, consisting of mouse α-chain and human β^S^-chain and human α-chain and mouse β-chain, which could exert a disease modification influence in steady state, and seems to act synergistically with quercetin reversing the H/R-induced severity of the disease. Accordingly, the recovery observed in the present study represents a combination of intrinsic anti-anemia therapeutic activity exerted by high oxygen affinity chimeric HbS with extrinsic antioxidant therapeutic activity of quercetin, to reverse the hypoxia induced severe disease pathology as well the steady state mild disease pathophysiology. Preliminary studies with transgenic knockout BERK mice models that do not produce high oxygen affinity chimeric HbS have shown significant improvements in disease pathology with quercetin treatment. However, they neither normalize the density gradient pattern of RBC, nor normalize the high CBF (cerebral blood flow) (data not shown), nor increase the hematocrit to normal levels with a single dose. Regarding the vaso-occlusion induced in wild type mice by H/R, even though it is low compared to NY1DD mice—the efficacy of quercetin to quench the vaso-occlusion is partial, despite the vaso-occlusion being limited. This is consistent with the novel concept of combination therapy to modify the SCD pathophysiology.

## Figures and Tables

**Table 1:** Effect of a single dose of quercetin (200 mg/kg, i.p.) on venular diameter, leukocyte adhesion, trans-endothelial emigration, and leukocyte rolling flux in transgenic sickle (NY1DD) mice subjected to H/R.

Parameter	Normoxia		Hypoxia Reoxygenation (NY1DD Mice)	
	Wild Type (C57BL/6J)	Sickle Mice (NY1DD)	Untreated	Quercetin (200 mg/kg)
Diameter (μm)	27.18 ± 0.99	25.66 ±0.81	24.76 ± 0.73	26.02 ± 1.24
Leukocyte adhesion (Cells/100 μm)	0.5 ± 0.15	1.7 ± 0.26 ^+^	5.8 ± 0.57 *	0.47 ± 0.12 ***
Leukocyte emigration (Cells/field)	0.11 ± 0.07	0.74 ±0.12 ^+^	1.7 ±0.28 *	0.22 ± 0.09 ***
Leukocyte rolling flux (Cells/min)	16.0 ± 5.11	36.71 ± 1.44 ^++^	47.17 ± 2.8 *	30.19 ±1.96 ***

Values represent the mean ± SEM (four animals from each group). Data were compared using one way-ANOVA followed by ^+^ *p* < 0.028; ^++^ *p* < 0.009 vs. normoxic wild type controls; * *p* < 0.0001 vs. respective normoxic wild type and NY1DD mice; *** *p* < 0.0001 vs. NY1DD mice subjected to H/R (untreated).

## Data Availability

Data obtained during the course of these studies in animals can be made available through contact with the corresponding author.
